# Children Affected by War and Armed Conflict: Parental Protective Factors and Resistance to Mental Health Symptoms

**DOI:** 10.3389/fpsyg.2017.01397

**Published:** 2017-08-23

**Authors:** Michelle Slone, Anat Shoshani

**Affiliations:** ^1^School of Psychological Sciences, Tel Aviv University Tel Aviv, Israel; ^2^Baruch Ivcher School of Psychology, Interdisciplinary Center Herzliya Herzliya, Israel

**Keywords:** resilience, armed conflict, parenting style, parental warmth, post-trauma

## Abstract

This study examined the role of parenting styles and parental warmth in moderating relations between exposure to political life events and mental health symptoms among 277 Israeli adolescents aged 12–14 and their parents, who had been exposed to protracted periods of war, missile bombardments, and terrorism. Adolescents completed the Political Life Events (PLE) scale, Brief Symptom Inventory and questionnaires regarding parenting style and parental warmth. The primary caregiver completed the Child Behavior Checklist for assessment of the child’s internalizing and externalizing symptoms. Results confirmed that severity of PLE exposure was positively correlated with psychological distress and with internalizing and externalizing symptoms. Maternal authoritativeness and warmth functioned as protective factors and had moderating effects on the relation between PLE exposure and mental health symptoms. In contrast, maternal authoritarianism exacerbated the relation between PLE exposure and children’s externalizing symptoms. Fathers’ parenting style and warmth had no significant relationship with children’s mental health outcomes. These findings have important clinical and practical implications for parental guidance and support during periods of war and armed conflict.

## Introduction

Children who grow up during war, armed conflict and terrorism experience dangerous events that threaten their mental health and normative age-related transitions. Growing up in these unstable and perilous environments is associated with psychological difficulties both among children and adolescents (Betancourt and Khan, 2008; Slone and Shoshani, 2008a). However, the wide individual differences shown in adjustment to these conditions are indicative of the many vulnerability and resilience factors that influence outcomes (Bonnano, 2004). Numerous factors have been studied as possible risk or resilience moderating factors ranging from children’s personal features and temperament to family and environmental characteristics (Slone and Shoshani, 2006; Shoshani and Slone, 2016). Despite the plausible role of parenting style in children’s adjustment to traumatic experiences (Baumrind, 1995; Steinberg, 2001), this aspect has been scarcely studied in the context of armed conflict and war. In view of this, the current study examined the moderating function of parenting style and parental warmth on the effects of children’s exposure to armed conflict and war. The study was conducted in Israel after a particularly traumatic period of missile attacks on residential areas that culminated in Operation Pillar of Defense which exerted an enormous toll on the entire country.

### Parenting Characteristics and Styles

Several studies have confirmed the central role of positive family interrelations and the ability to confront trauma as a cooperative unit in mitigating children’s negative reactions (Punamäki et al., 2011). Parental love and family intimacy have been shown to assist children muster their competencies and to increase coping among families that endure war and armed conflict (Punamäki et al., 2001). Family cohesion and provision of a sense of security increase resilience among children exposed to political conflict (Laor et al., 1997) especially in combination with maintenance of a familiar routine (Pat-Horenczyk et al., 2006). Children with positive perceptions of parental protection, support and monitoring frequently overcome the traumatic events involved in political conflict with no decline in mental health or functioning (Barber, 1999). This evidence for the important role of familial factors in moderating children’s symptoms after stressful exposure provides a compelling rationale for examining the function of parenting style and parental warmth in mitigating children’s post-trauma reactions.

The seminal definition of parenting style refers to ways in which parents exercise control and respond to their children and is conceptualized as varying along two orthogonal dimensions of responsiveness and demandingness (Baumrind, 1991). In combination, these two dimensions yield three styles. The *Authoritarian Parenting Style*, defined as low in responsiveness and highly demanding, is characterized by strict control and limited emotional support. Authoritarian style is reflected in stringent appraisal of the child’s attitudes and behavior according to strict criteria of conduct compiled by a higher authority. This interprets into restriction of the child’s autonomy, demands for obedience, and stringent control over the child’s self-will (Baumrind, 1966, 1968). The *Authoritative Parenting Style* is defined as employing a balance of responsiveness with demandingness and is characterized by the supply of emotional support together with firm but responsive discipline. This style manifests in firm control at times of discord balanced with guidance, explanation, flexibility, openness to negotiation, tolerance and acceptance of individuality. Thirdly, the *Permissive Parenting Style*, manifests high levels of responsiveness to the child and low levels of demandingness. This style is exhibited in generous provision of support, encouragement, independence and autonomous decision-making with a scarcity of control, supervision and discipline (Baumrind, 1991). Studies of the effects of the different parenting styles have consistently indicated the dominance of the authoritative style in supporting positive adjustment of children (Baumrind, 1989; Park and Bauer, 2002). In contrast, the authoritarian and permissive parenting styles have been associated with poor adjustment (Baumrind, 1989; Park and Bauer, 2002). The authoritarian style has been connected to difficulties in intimate relationships (Sharabany et al., 2008), low self-esteem, decision-making difficulties and depressed mood (Wenar and Kerig, 2000). The permissive style has been associated with less successful socialization and low self-esteem (Baumrind, 1991), impulsiveness, antisocial behavior and lack of empathy (Wenar and Kerig, 2000). However, this evidence is far from conclusive and the role of different parenting styles in children’s adjustment to trauma has not been systematically investigated in contexts of chronic political violence exposure.

### Parental Warmth and Child Adjustment

Parent–child relational quality is an important predictor of positive developmental outcomes and adjustment (Cox and Harter, 2003). An affectionate and empathic parent–child relationship can protect against stress during times of war (Garbarino, 1992; Bat-Zion and Levy-Shiff, 1993). Parental compassion reflected in holding and containment are associated with effective coping among children whereas parental expressions of anger and exasperation are linked to heightened emotional distress and behavioral difficulties among children (Bat-Zion and Levy-Shiff, 1993). Parental warmth has been described as the expression of interest and involvement in children’s activities, encounters and peer relations, displays of enthusiasm for children’s achievements, and demonstrations of affection and love (Amato, 1990). Parental warmth reduces emotional distress (Operario et al., 2006) and the propensity for dangerous behavior among adolescents (Nash et al., 2005) and promotes healthy psychosocial development (Steinberg, 2001). Parental warmth may serve as a buffer between negative ecological and familial factors such as family economic hardships, high-risk neighborhoods and stressful life experiences, and individual outcomes (Kim et al., 2003). However, the protective function of parental warmth may vary across the racial, ethnic and cultural diversity found among family systems (Jaggers et al., 2017).

Parenting styles and parental warmth can play an important role in the lives of children who are traumatized by war and armed conflict, warranting examination of their role as a source of resilience. Despite the centrality of parental warmth and parenting style in children’s development, surprisingly few studies have examined their specific protective role in the context of political violence.

The present study examined the moderating role of parental practices and warmth on children’s mental health symptoms operationalized as psychological distress and internalizing and externalizing symptoms. Internalizing symptoms are characterized by developmentally inappropriate problems of excessive withdrawal, anxiety, depression, and psychosomatic complaints. Externalizing symptoms refer to inappropriate, maladaptive externally directed behaviors that frequently exhibit in excessively impulsive, delinquent or aggressive behavior (Achenbach and Edelbrock, 1981).

The mass of findings showing ways that parenting practices can exert profound effects on children’s mental health and behavior point to some specific relations between parenting styles and parental warmth and children’s internalizing and externalizing symptoms. In general, cold, harsh, and inconsistent parenting has been found to increase the risk for both externalizing and internalizing disorders and conversely, parental monitoring has been shown to predict better adjustment among adolescents (Roche and Leventhal, 2009) and parental supervision has emerged as a significant predictor of less aggression, less oppositional and violent behavior and less risk-taking behavior behaviors among adolescents (Vazsonyi et al., 2006). Several mechanisms have been postulated to account for these relations. Strict parental control tactics may imply rejection and disregard of the adolescent’s views, needs and choices (Rogers et al., 2003). Harsh demands for obedience without explanation could impair the child’s autonomy-seeking and self-competence and increase avoidance and dependence (McLeod et al., 2007; Murray et al., 2009).

In addition to breaking down the relations between specific parental practices and parenting warmth and internalizing and externalizing mental health symptoms, the present study supplements existing research in several further directions. Findings could provide important information about patterns of parenting styles in populations not previously reported. Further, the present study aimed to examine the moderating role of parenting style between severe trauma exposure and children’s mental health symptoms, thus moving beyond a correlational description toward investigations of process. This is relevant beyond the specific geo-political context of this study since it could contribute to our knowledge of the functioning of resilience factors in the lives of children who develop in environments of war and conflict.

### Effects of Exposure to Armed Conflict on Children

The protracted conflict between Israel and the surrounding Arab states and populations has taken many forms including wars, prolonged episodes of hostility, terrorism and missile attacks. These hostilities have resulted in destruction, damage, fatalities and injuries among all parties (Golan and Shai, 2004). The most recent phase of the conflict has been concentrated on hostilities between the Israelis and Palestinians in the vicinity of Gaza. Gaza has suffered numerous military operations and attacks and infrastructure has been largely damaged by a blockade on all borders. Israel has suffered periods of terrorism and intense rocket and missile bombardments that began in the south of the country, but more recently occurring with an expanding perimeter of long-range missile attacks into central and parts of northern Israel as well. At the sounding of the alarm, inhabitants of cities and towns within these regions have between 15 s and 1 min to find shelter.

The present study was conducted in 2013 among families in southern Israel a few weeks after Operation Pillar of Defense. Operation Pillar of defense was an 8-day Israeli operation in the Gaza Strip which commences on November 14, 2012. For the Israeli population, the operation was preceded by an estimate of 2221 rockets and 196 mortars fired in 2012 and a period with a number of mutual Israeli–Palestinian responsive attacks (Zucker and Kaplan, 2014). Thus, since exposure in chronic conflict in cumulative, the families and children involved have been exposed to a wide variety of stressful and traumatic events related to the intractable conflict (Slone and Shoshani, 2014).

Growing up in conditions of armed conflict, terrorism and war has been associated with a variety of negative psychological consequences, particularly post-traumatic stress (PTS) symptoms and a spectrum of overt and covert symptoms and disorders (Betancourt and Khan, 2008; Slone and Shechner, 2009). Short and long-term effects have been found with short-term effects including distress, shock, fear, anger (Slone et al., 2008; Shoshani and Slone, 2008a,b) and aggressive behavior (Guttmann-Steinmetz et al., 2011). Findings for long-term effects is inconsistent showing mixtures of increased manifestation of externalizing disorders (Barber, 2001; Muldoon, 2004), sub-clinical symptoms (Slone and Shoshani, 2008b) through to anxiety and depression (Gupta and Zimmer, 2008; Slone and Shoshani, 2010) and PTS and full-blown PTSD (Finzi-Dottan et al., 2006).

In view of this, the present study examined the moderating function of the three parenting styles, authoritarian, authoritative and permissive, and parental warmth for children exposed to war and political conflict. The moderating role of parenting style and parental warmth was examined among families living in southern Israel which had suffered extended periods of missile attacks and other forms of conflict-related violence several weeks after a traumatic military offensive. The study included assessment of severity of exposure to traumatic political conflict events, assessment of relations between trauma exposure and mental health symptoms, and examination of the moderating function of the three parenting styles and parental warmth in relations between exposure and outcome.

#### Hypotheses

In line with the rationale proposed, the present study examined three hypotheses. The first hypothesis predicted a direct relation between political violence exposure and children’s mental health symptoms, such that high levels of exposure would be related to higher levels of mental health symptoms. The second hypothesis predicted a moderating effect of parental styles on the relations between political violence exposure and mental health symptoms according to which the authoritative style, but not the authoritarian and permissive styles, would be a protective factor against mental health symptoms especially at high levels of PLE exposure. The third hypothesis predicted that parental warmth would moderate the relation between political violence exposure and the child’s mental health symptoms. The difference in the level of the child’s internalizing, externalizing symptoms and general distress between high and low exposure will be greater for children with low parental warmth than for children with high parental warmth.

## Materials and Methods

### Participants

Participants were 277 seventh and eighth grade adolescents (136 girls, 141 boys) aged 11.9 to 14.1 (*M* = 12.94, *SD* = 0.75) from two middle schools located in the southern Israeli city of Ashkelon and their parents. The majority of the study population reported middle-class socio-economic (57%) and others reported low (23%) and high socio-economic status (20%). The majority of students were Jewish (96%), with 3% reporting Orthodox adherence, 31% traditional and 66% secular. The parental questionnaire was completed by the primary caregiver 88% being mothers. The age range of the parents was 32–46 years (*M* = 42.9, *SD* = 4.87). Marital status of parents was reported as 1% single, 94.4% married and 4.6% divorced.

### Measures

#### Child Report

##### Political Life Events Scale (PLE) (Slone et al., 1998)

The PLE scale contains 20 items representing events to which participants could have been exposed. Participants were requested to record whether they had been exposed to each of the events over the past year. The PLE scale yields a severity of exposure score that is calculated by summing all items marked for exposure, weighted on the basis of previously determined evaluations of the severity of items. The formula for items severity is: mild items (e.g., security check on entering a public place) are multiplied by 1, moderate items (e.g., being in the vicinity of an explosion or missile attack) are multiplied by 2, and severe items (e.g., injury to a family member as a result of war or military circumstances) are multiplied by 3. The PLE scale has been used widely both in Israel and internationally and has shown high predictive validity for communities exposed to armed conflict and war (Slone et al., 2009). Test–retest scores range from *r* = 0.86 to *r* = 0.94 (Slone and Shechner, 2009). There is empirical support for the distinctiveness of the PLE from general life events scales (Slone and Roziner, 2013). This study yielded a Cronbach’s alpha coefficient of 0.92.

##### Brief Symptom Inventory (BSI) (Derogatis and Spencer, 1982)

The Brief Symptom Inventory is the abbreviated version of the SCL-90-R and consists of 53 self-report items rated on a scale from 0 (not at all) to 4 (very much). This measure provides distress indices and assessment of symptom load across 10 dimensions-somatization, obsessive-compulsive, interpersonal sensitivity, depression, anxiety, hostility, phobic anxiety, paranoid ideation, psychotic ideation and one miscellaneous symptom subscale. The BSI has yielded good reliability and validity measures (α = 0.71–0.81), high test – retest reliability (correlations between 0.78 and 0.90). The inventory has been back-translated into Hebrew with good internal consistency and concurrent validity (Canetti et al., 1994). As a single summary measure, the Global Severity Index (GSI) reflects depth of general psychological distress irrespective of the specific symptoms from which the distress arises and is reported to be the best indicator of depth of distress (Derogatis and Spencer, 1982). The GSI score is calculated as the average of ratings assigned to the presence of each symptom. In the current study, the BSI yielded a Cronbach’s alpha coefficient of 0.89.

##### Parental Acceptance Rejection Questionnaire (PARQ) (Rohner, 1986)

Parental warmth was measured by the warmth/affection subscale of the Parental Acceptance Rejection Questionnaire (PARQ; Rohner, 1986). Children were administered two questionnaires, one relating to father and one relating to mother. The subscale used contained 20 items rated on a 6-point Likert scale of 1 (almost never true) to 6 (almost always true). Test–retest reliability of the instrument has yielded a correlation of 0.62 for time periods of 3 weeks to 7 years. In the present study, internal consistency was good with a Cronbach’s alpha coefficients of 0.80 for reports on fathers and 0.85 for reports on mothers.

##### Parental Authority Questionnaire (PAQ) (Buri, 1991)

The Parental Authority Questionnaire consists of 30 items reflecting the three parenting styles with 10 items for the permissive, 10 items for the authoritarian, and 10 items for the authoritative style. Participants rate items for agreement on a five-point Likert scale ranging from strongly disagree (1) to strongly agree (5). Each of the statements is based on Baumrind’s (1971) definitions of permissive, authoritarian, and authoritative parental prototypes. As an example, the permissive scale contains the following item: “My mother/father has always felt that what children need is to be free to make up their own minds and to do what they want to do, even if this does not agree with what the parents might want.” An example of an item from the authoritarian scale is: “As I was growing up my mother/father did not allow me to question any decision that she/he had made.” An example of an item from the authoritative scale is: “My mother/father has always encouraged verbal give-and-take whenever I have felt that family rules and restrictions were unreasonable.” Each adolescent completed two forms of the PAQ, one referring to the parenting style characteristic of the mother and one referring to that characteristic of the father. Buri (1991) has reported acceptable test–retest reliabilities over a 2- week period (ranging from 0.77 to 0.92) and internal consistency (Cronbach’s alpha coefficients ranging from 0.74 to 0.85) for the different scales. In this study, Cronbach’s alpha coefficients ranged from 0.72 to 0.88.

#### Parental Report

##### Child Behavior Checklist (CBCL) (Achenbach and Edelbrock, 1981)

Children’s affective and behavioral difficulties were assessed using the extended version of the Age 4–18 Form of the CBCL (Achenbach and Edelbrock, 1981). Children’s primary caregivers were requested to note the presence of their children’s behaviors or affect on items on a scale ranging from 0 (not true of the child) to 1 (somewhat true of the child) to 2 (often true or very true of the child). The CBCL is one of the most extensively used instruments for psychopathological assessment in both research and clinical practice with children. The internalizing scale is represented by summing 32 items that load into three clinical syndrome scales: Withdrawn (9 items) for example “Complains of loneliness,” Somatic Complaints (9 items) for example “Has stomach aches with no known medical cause,” and Anxious/Depressed (14 items) for example “Too fearful or anxious.” The externalizing scale is represented by summing 27 items from two clinical syndrome scales: Delinquent Behavior (8 items) for example “Breaks rules at home, school and elsewhere” and Aggressive Behavior (19 items) for example “Gets into fights a lot.” One-week test–retest stability coefficients are reported as 0.89 for internalizing problems and 0.93 for externalizing problems (Achenbach and Edelbrock, 1981). In this study, Cronbach’s alpha coefficients were 0.91 for the internalizing scale and 0.93 for the externalizing scale.

### Procedure

After receiving authorization from the Tel Aviv University Ethics Committee and the Israeli Ministry of Education, researchers randomly selected 350 families from classroom lists of schools. Access to parents was authorized by the Israeli Ministry of Education and the schools under restrictions of use only for research purposes, preservation of confidentiality, preservation of anonymity of participants in research findings, and allowance of termination of participation in the study at will of participants. Families were approached by a member of the research team who explained the research and determined willingness to participate in the study. A total of 277 of the parents and adolescents signed informed consent to participate in the study. Questionnaires were administered to the child in a quiet room in the child’s home and the primary caregiver was requested to complete the CBCL alone and to transfer it to the researcher in a sealed envelope. Families were compensated for their time with a $15 voucher.

### Data Analysis

Data were analyzed using SPSS 21.0. In order to examine the effects of Political Life Events (PLE), parenting styles and parental warmth on mental health symptoms and the moderating effects of parenting styles and parental warmth on the relation between PLE and mental health, three hierarchical (three steps) linear regression analyses were computed. The GSI (Global Severity Index of the Brief Symptom Inventory), externalizing symptoms and internalizing symptoms were entered as the dependent variables, one in each regression. The main effect of PLE was examined in the first block, parenting styles and parental warmth were included in the second block, and interaction effects between the PLE and the parenting variables were examined in the third block.

Simple effects of the significant interactions from this set of analyses were then examined, using the PROCESS macro for SPSS (Hayes, 2013), a computational macro for path analysis-based moderation. The mean ± 1 standard deviation of the moderating variables were used to calculate their conditional effects (Hayes, 2013). The SPSS Missing Value Analysis module was used to estimate the pattern of missing data. Missing data comprised less than 3% of the total data and the missing values were replaced using a multiple imputation method (Tabachnick and Fidell, 2007).

## Results

### Preliminary Data Analyses and Descriptive Statistics

As a preliminary step, tests of kurtosis and skewness were conducted to verify the normality of the study variables. The absolute values of kurtosis and skewness were relatively small and therefore no data transformations were made. Preliminary *t*-test and bivariate correlation analyses showed no significant effects of children’s age and gender on the study variables. The means, standard deviations, and correlations of the study variables are presented in **Table 1**.

**Table 1:** Means, standard deviations and bivariate correlations for the study variables.

	Mean	*SD*	2	3	4	5	6	7	8	9	10	11	12
1. PLE	8.41	4.08	0.55^∗∗∗^	0.34^∗∗∗^	0.45^∗∗∗^	–0.05	–0.09	0.01	–0.07	0.06	–0.01	–0.07	–0.11
2. GSI	0.79	0.53	–	0.33^∗∗∗^	0.41^∗∗∗^	–0.09	–0.53***	0.01	–0.03	0.10	0.01	0.04	–0.08
3. Internalizing symptoms	5.30	5.17		–	0.64^∗∗∗^	–0.07	–0.35***	–0.03	0.04	0.01	–0.03	0.32***	0.01
4. Externalizing symptoms	5.68	6.11			–	–0.12	–0.51***	0.01	–0.09	0.09	–0.01	0.32***	–0.06
5. Paternal warmth	5.35	0.44				–	0.13*	0.17**	–0.07	0.03	0.08	–0.11	–0.08
6. Maternal warmth	5.27	0.70					–	0.13*	–0.09	–0.22**	0.09	–0.17**	0.01
7. Paternal authoritativeness	3.94	0.50						–	–0.01	–0.01	0.18**	0.02	–0.09
8. Paternal authoritarianism	2.71	0.68							–	0.03	–0.07	0.09	0.02
9. Paternal permissiveness	2.47	0.53								–	–0.07	0.07	0.08
10. Maternal authoritativeness	4.11	0.46									–	–0.07	0.07
11. Maternal authoritarianism	2.69	0.71										–	0.06
12. Maternal permissiveness	2.45	0.55											–

*PLE, political life events; GSI, general severity index of the brief symptom inventory. ^∗^*p* < 0.05, ^∗∗^*p* < 0.01, ^∗∗∗^*p* < 0.001*; N* = 277.*

### Effects of PLE and Parenting Styles on Mental Health Symptoms

The first hierarchical regression analysis investigated the moderating effects of parenting styles and parental warmth on the relation between PLE and the GSI (Global Severity Index of the Brief Symptom Inventory). Block 1, that investigated the main effect of PLE, accounted for 24% of the variance in the GSI. Greater severity of political life events was associated with higher GSI levels (β = 0.49, *p* < 0.001). The second block of the regression, including the PLE together with the parenting variables, accounted for an additional 15% of the total variance. Maternal warmth was negatively associated with GSI levels (β = -0.51, *p* < 0.001). The other parenting characteristics (mothers’ and fathers’ parenting styles and paternal warmth), did not predict significantly the GSI levels. The third block of the regression, including the addition of the two-way interactions between the PLE and the parenting variables to the model, accounted for 1.3% of the total variance in the GSI, *R*^2^ = 0.40, *F*(17,259) = 7.77, *p* < 0.001. However, none of the two-way interactions was statistically significant (see **Table 2**).

**Table 2:** Multivariate regression analyses predicting mental health symptoms.

	GSI			Internalizing symptoms			Externalizing symptoms		
	*B*	*SE*	β	*B*	*SE*	β	*B*	*SE*	β
**(Constant)**	–0.03	0.06		0.02	0.06		–0.08	0.05	
**Block 1**									
PLE	0.27	0.06	0.29***	0.24	0.07	0.24***	0.17	0.05	0.19***
**Block 2**									
Paternal warmth	–0.03	0.06	–0.03	0.03	0.07	0.02	0.07	0.05	0.07
Maternal warmth	–0.55	0.10	–0.51***	–0.33	0.11	–0.29***	–0.46	0.08	–0.44***
Paternal authoritativeness	0.06	0.06	0.06	–0.04	0.06	–0.04	0.00	0.05	0.01
Paternal authoritarianism	–0.10	0.07	–0.10	–0.01	0.07	–0.01	–0.03	0.06	–0.03
Paternal permissiveness	–0.10	0.07	–0.10	–0.14	0.07	–0.11	–0.06	0.06	–0.06
Maternal authoritativeness	0.00	0.06	0.00	0.02	0.06	0.02	0.02	0.05	0.02
Maternal authoritarianism	0.03	0.06	0.03	0.28	0.07	0.27***	0.23	0.05	0.25***
Maternal permissiveness	0.02	0.06	0.02	0.09	0.07	0.08	0.01	0.05	0.02
**Block 3**									
Paternal warmth × PLE	–0.08	0.07	–0.09	–0.07	0.07	–0.06	0.04	0.06	0.04
Maternal warmth × PLE	0.07	0.05	0.13	–0.01	0.05	–0.01	–0.10	0.04	–0.13*
Paternal authoritativeness × PLE	–0.01	0.07	–0.01	–0.05	0.07	–0.04	–0.01	0.06	–0.01
Paternal authoritarianism × PLE	0.00	0.08	0.00	–0.16	0.09	–0.14	–0.02	0.07	–0.02
Paternal permissiveness × PLE	–0.07	0.08	–0.06	0.01	0.09	0.00	–0.01	0.07	–0.01
Maternal authoritativeness × PLE	0.01	0.07	0.01	–0.22	0.07	–0.17**	–0.16	0.06	–0.16**
Maternal authoritarianism X PLE	0.01	0.08	0.01	0.05	0.08	0.05	0.13	0.06	0.13*
Maternal permissiveness × PLE	0.06	0.06	0.07	0.00	0.07	0.00	–0.02	0.05	–0.03
*R*^2^ for model 1	0.24***			0.15***			0.19***		
Δ*R*^2^ for model 2	0.15***			0.22***			0.32***		
Δ*R*^2^ for model 3	0.01			0.04			0.02		
*F* for model 3	7.77***			8.37***			13.68***		

*PLE, political life events; GSI, general severity index of the brief symptom inventory. ^∗^*p* < 0.05, ^∗∗^*p* < 0.01, ^∗∗∗^*p* < 0.001.*

The second hierarchical regression analysis examined the associations between the same predictors with children’s internalizing symptoms as the dependent variable. Block 1, consisting of the PLE main effect, accounted for 15% of the variance in the internalizing symptoms. Similar to the first analysis, PLE was positively associated (β = 0.39, *p* < 0.001) with internalizing symptoms. Block 2 that included the parenting variables accounted for an addition of 22% of the total variance. Maternal warmth was negatively associated (β = -0.29, *p* < 0.001) and maternal authoritarianism was positively associated (β = 0.27, *p* < 0.001) with levels of internalizing symptoms.

The third block of variables, accounted for an additional 4% of the variance in the internalizing symptoms, *R*^2^ = 0.41, *F*(17,259) = 8.37, *p* < 0.001. The two-way interaction of maternal authoritativeness × PLE was significant (β = -0.17, *p* = 0.003), whereas the other interactions were not statistically significant (**Table 2**).

Similar trends were found in the third regression analysis that examined relations between PLE, parenting styles and parental warmth on externalizing symptoms. PLE in the first block predicted 19% of the variance in the externalizing symptoms (β = 0.44, *p* < 0.001). The second block of the regression accounted for an additional 32% of the total variance in the externalizing symptoms with a negative relation for maternal warmth (β = -0.44, *p* < 0.001) and a positive relation for maternal authoritarianism (β = 0.25, *p* < 0.001). In the third block, the maternal authoritativeness × PLE interaction reached significance (β = -0.16, *p* = 0.002) as well as the maternal authoritarianism × PLE interaction (β = 0.13, *p* = 0.04) and the maternal warmth × PLE interaction [β = -0.13, *p* = 0.04), *R*^2^ = 0.54, *F*(17,259) = 13.68, *p* < 0.001]. In the three regression analyses, fathers’ parenting variables were not found to be significant predictors of mental health symptoms (see **Table 2**).

### Moderation Effects of Maternal Parenting Styles and Warmth

**FIGURE 1:**
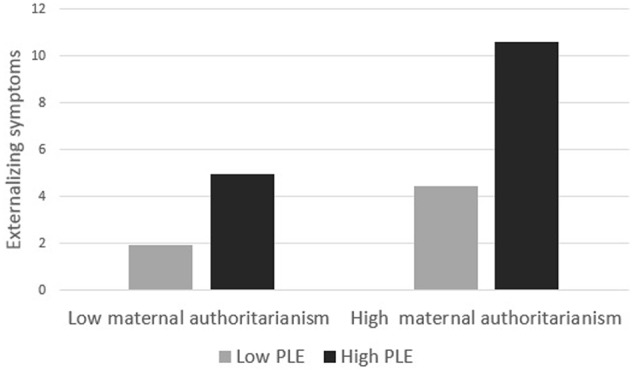
Externalizing symptoms as a function of political life events (PLE) and maternal authoritarianism.

In order to clarify the source of the significant interactions in the regression analyses, follow-up simple effects analyses were conducted, using the PROCESS macro for SPSS (Hayes, 2013). First, we examined the interaction between PLE and maternal authoritarianism on externalizing symptoms. In order to clarify the source of this interaction, we examined the externalizing symptoms as a function of PLE and maternal authoritarianism at 1 standard deviation above and below the mean (PLE: *M* = 8.41, *SD* = 4.07; maternal authoritarianism: *M* = 2.69, *SD* = 0.71) (Cohen and Cohen, 1983). As seen in **Figure 1**, for low maternal authoritarianism, there were significant differences in externalizing symptoms between children with low PLE (*M* = 1.93) and high PLE (*M* = 4.93), with high PLE associated with higher levels of symptoms *(b* = 0.25, *SE* = 0.07, *t* = 3.32, *p* = 0.001). However, for high maternal authoritarianism, these differences became more pronounced for participants with high PLE (*M* = 10.6) compared to participants with low PLE (*M* = 4.43). Thus, high maternal authoritarianism exaggerated the effects of PLE on the externalizing symptoms (*b* = 0.50, *SE* = 0.08, *t* = 6.41, *p* < 0.001).

**FIGURE 2:**
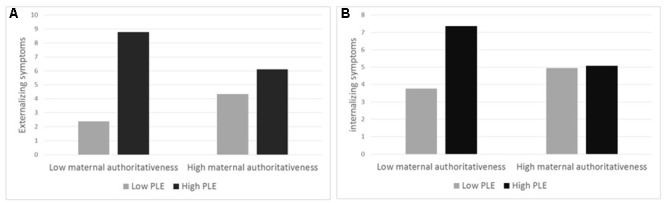
The interaction effect that emerged between PLE and maternal authoritativeness both on externalizing symptoms **(A)** and internalizing symptoms **(B)**.

Another interaction effect emerged between PLE and maternal authoritativeness on both externalizing symptoms and internalizing symptoms. As shown in **Figure 2A**, tests of simple effects revealed that for low maternal authoritativeness, children exhibited low levels of externalizing symptoms at low PLE (*M* = 2.38), but their symptoms increased dramatically at high PLE (*M* = 8.78, *b* = 0.52, *SE* = 0.09, *t* = 5.90, *p* < 0.001). In contrast, the effect of PLE on externalizing symptoms was not significant for high maternal authoritativeness (*b* = 0.14, *SE* = 0.09, *t* = 1.64, *p* = 0.10), as there were no significant differences in externalizing symptoms at low (*M* = 4.33) or high PLE (*M* = 6.1). This indicates that maternal authoritativeness moderated the increase in externalizing symptoms. A similar trend was observed for internalizing symptoms (see **Figure 2B**), as PLE had a significant effect on children’s internalizing symptoms for low maternal authoritativeness (*b* = -0.35, *SE* = 0.10, *t* = 3.47, *p* < 0.001), but not for high maternal authoritativeness (*b* = -0.01, *SE* = 0.10, *t* = 0.01, *p* = 0.98).

**FIGURE 3:**
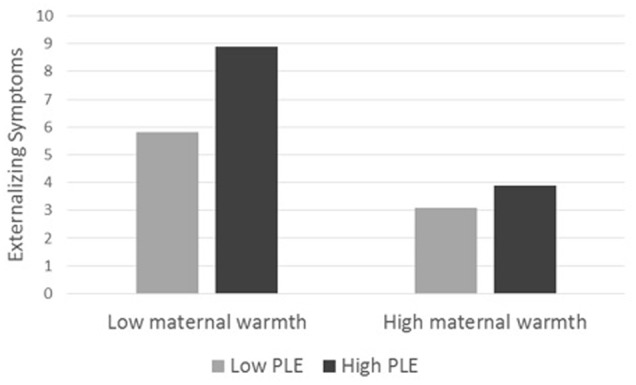
Externalizing symptoms as a function of PLE and maternal warmth.

In addition, a significant interaction effect was found between maternal warmth and PLE for externalizing symptoms, as maternal warmth moderated the increase in externalizing symptoms. Tests of simple effects revealed that for low maternal warmth, there were significant differences in externalizing symptoms between children with low PLE (*M* = 5.80) and high PLE (*M* = 8.89, *b* = 0.25, *SE* = 0.06, *t* = 4.07, *p* < 0.001). However, the effect of PLE on externalizing symptoms was not significant for high maternal warmth (*b* = 0.06, *SE* = 0.08, *t* = 0.87, *p* = 0.38), as there were no significant differences in externalizing symptoms which remained low at both low (*M* = 3.08) and high PLE (*M* = 3.90) (see **Figure 3**).

## Discussion

This study examined the effects of parental features on children’s mental health symptoms during traumatic periods of war and armed conflict. Specifically, the study focused on the protective function of parenting styles and parental warmth in mitigating children’s symptoms on the assumption that parents hold a critical role in regulating and containing children’s post-traumatic symptoms after exposure to hostilities and war.

The first hypothesis predicting a direct positive relation between level of exposure to traumatic events and mental health symptoms was confirmed. Greater severity of exposure was associated with more severe internalizing and externalizing symptoms and general psychological distress. This finding confirms the dose-response effect that refers to the relationship between the magnitude of a stressor to the response of the receptor. The dose-response effect for traumatic exposure would predicate exacerbation of negative reactions to increasing levels of traumatic experiences (Braun-Lewensohn et al., 2009) as confirmed by our findings. Interestingly, at high PLE both internalizing and externalizing symptoms and general psychological distress were elevated, demonstrating the wide variation in children’s psychological response to these threatening and dangerous experiences. Surprisingly, no gender differences emerged in the relation between exposure and the various symptoms despite traditional research findings showing greater internalizing symptoms among females and more severe externalizing symptoms among males as a result of exposure to stress (Leadbeater et al., 1999). Possibly, the severity and intensity of exposure to war eradicates these gender differences and highlights the multi-domain response of children to the trauma of war and armed conflict.

The second hypothesis predicted a moderating effect of parental styles on the relations between political violence exposure and mental health symptoms. This hypothesis was partially confirmed with a complex combination of findings. No effect emerged for fathers’ parenting style on any of the outcome variables. In contrast, mothers’ parenting style significantly moderated the relation between children’s level of exposure and internalizing and externalizing symptoms, but not general psychological distress. In the case of the authoritative parenting style, the relation between children’s level of exposure to political life events and internalizing and externalizing symptoms was not significant in families in which children perceived their mothers to be highly authoritative. However, children’s perception of mothers’ low authoritativeness was associated with more severe internalizing and externalizing symptoms at high levels of political violence exposure.

The authoritative parenting style contains two elements that could be relevant for emotional regulation after trauma exposure. High responsiveness and empathic support in the family have been identified as powerful resilience factors for children in contexts of war, armed conflict and terrorism (Slone et al., 2009). In addition, the component of demandingness based on negotiation and dialog could provide a holding space that encourages self-control and structure after chaotic and traumatic times. These findings present compelling evidence for the protective function of maternal authoritative parenting style when children are exposed to these highly traumatic experiences.

These effects emerged only for internalizing and externalizing symptoms and not for general psychological distress as measured by the BSI. This finding could result from the different informants responding to the questionnaires since the primary caregiver completed the CBCL and the child completed the BSI. In addition, the two scales reflected different symptoms profiles. The CBCL reflected parent reports of children’s behaviors and impressions of mood. The BSI assesses a spectrum of psychiatric clusters derived from the presence of psychiatric symptoms such as Obsessive Compulsive Disorder and paranoid ideation which may reflect more severe difficulties beyond those that could be explained as influenced by parenting styles.

In the case of the authoritarian parenting style, mothers’ high authoritarianism exacerbated the relation between severity of exposure and children’s externalizing symptoms, but not internalizing symptoms or general psychological distress. This finding suggests that highly demanding and strict control together with low responsiveness is particularly onerous for children exposed to trauma. It is possible that the multiple burdens of negotiating early adolescence together with traumatic experiences in a highly demanding, non-supportive and under-responsive family environment can prompt the child to act out with increased behavioral difficulties.

The third hypothesis predicting a moderating effect of parental warmth on the relation between PLE and the outcome variables was confirmed only for maternal warmth on children’s externalizing symptoms. Among children who perceived their mothers as very warm and affectionate, the relation between children’s level of political violence exposure and externalizing symptoms was not significant. However, low maternal warmth was associated with increased externalizing symptoms with severe political violence exposure. This finding concurs with identification of the important role of parents’ acceptance, support and containment of children during war and armed conflict (Laor et al., 1997; Pat-Horenczyk et al., 2006) and distills the particular and unique function of maternal nurturing and warmth in soothing children’s reactions to trauma.

It is important to note the surprising non-significant findings for fathers’ parenting styles and warmth in moderating the effects of trauma exposure on children’s mental health symptoms. This concurs with other findings conducted in Israel showing that paternal parenting style was not associated with children’s level of psychological distress (Slone et al., 2011). This was suggested as indicating the dominant influence of mothers in the family in the particular research population, particularly with regards to children’s emotional well-being. Our finding of lack of significance of paternal parenting style on children’s mental health symptoms could accord with this explanation. In the specific case of the current study, the primary caregiver in the family was documented since they were requested to complete the parent report on the child’s symptoms. In our sample, 88% of mothers were reported as the primary caregiver. This suggests that in our particular sample and at the particular post-war administration period of this study mothers held a dominant role and this could possibly account for the salience of maternal effects on children’s outcomes.

### Limitations and Implications

Despite the interesting evidence for the moderating role of parenting styles and warmth yielded here, this study had several noteworthy limitations. In this study, children reported on their perceptions of parenting style and warmth. This should be supplemented with parent report of these measures. The findings cast light on aspects of the role of family functioning in a post-war period during which parents and children may have been particularly traumatized and vulnerable. As such, the findings have particular clinical implications for enhancing children’s resilience in post-traumatic circumstances. Practitioners and psychologists treating children should be aware of the crucial role of maternal warmth in children’s lives. This role is expressed by the fact that, regardless of other parental practices, a warm, caring and loving family environment is directly associated with children’s resilience in traumatic life circumstances.

It is important to note that these findings may not be generalizable across different traumatic circumstances and for long-term outcomes. Children’s long-term needs after exposure to trauma may be very different from immediate or short-term emergence from traumatic circumstances. In addition, the characteristics of parenting style and warmth could be particular to the actual stressful period and may reflect the parents’ own stress rather than generic parenting style at different periods. Therefore, longitudinal research that follows fluctuations in parenting practices and children’s symptoms would provide a broader picture of the family role in children’s adjustment to trauma.

Maternal authoritative parenting style and warmth emerged as potent protectors against children’s mental health symptoms. The components of this parenting style highlight the importance of negotiation and dialog in control practices, respect, support, affection in responsiveness, and warm interrelationships for children’s safe passage through traumatic circumstances. This could be a challenge for stressed and traumatized parents. The complexity of balancing demandingness, responsiveness and warmth may be particularly pertinent in families of early adolescents who are themselves encountering the growth in maturity to achieve self-autonomy and consolidate identities. Nonetheless, knowledge of the importance of these components can guide psychologists and counselors who are faced with the task of accompanying families through traumatic circumstances and experiences.

## Ethics Statement

This study was carried out in accordance with the recommendations of the Tel Aviv University Ethics Committee and the Israeli Ministry of Education with written informed consent from all subjects. All subjects gave written informed consent in accordance with the Declaration of Helsinki. The protocol was approved by the Tel Aviv University Ethics Committee.

## Author Contributions

All authors listed have made a substantial, direct and intellectual contribution to the work, and approved it for publication.

## Conflict of Interest Statement

The authors declare that the research was conducted in the absence of any commercial or financial relationships that could be construed as a potential conflict of interest. The reviewer YL declared a shared affiliation, though no other collaboration, with one of the authors MS to the handling Editor, who ensured that the process nevertheless met the standards of a fair and objective review.
